# Supplementary material for: Comparative Analysis of Chloroplast Genome in Saccharum spp. and Related Members of ‘Saccharum Complex’
Source: Int J Mol Sci. 2022 Jul 11;23(14):7661. doi: 10.3390/ijms23147661 (PMC9315705; doi:10.3390/ijms23147661)
Supplement: Supplementary file 1 [file ijms-23-07661-s001.zip › ijms-1783669-supplementary/supplementary_files/Table S1&S2.pdf]

Table S1. The List of *Saccharum* and relative species included in this study.

Species	Sample	Region	Description
S. spontaneum	SP79-9	Guangxi, China	Firstly sequenced and assembled in this study
	IND84-066	Coimbatore, India	From NCBI SRA database and assembled in this study
	SES14	Coimbatore, India	From NCBI SRA database and assembled in this study
	IND81-013	Coimbatore, India	From NCBI SRA database and assembled in this study
S. robustum	NG57-208	Papua New Guinea	Firstly sequenced and assembled in this study
	NG51-63	Papua New Guinea	Firstly sequenced and assembled in this study
	IN84-076	Baini, Sulawesi Tenggara, Indonesia	Firstly sequenced and assembled in this study
	IS76-184	Palopo, South Sulawesi, Indonesia	Firstly sequenced and assembled in this study
S. sinense	Guilinzhuzhe	Guangxi, China	Firstly sequenced and assembled in this study
	Youba	China	Firstly sequenced and assembled in this study
S. barberi	Katha	India	Firstly sequenced and assembled in this study
	Chunne	India	From NCBI SRA database and assembled in this study
	Kewali	India	Firstly sequenced and assembled in this study
S. officinarum	Loether	Mexico	Firstly sequenced and assembled in this study
	Blackcheribon	Hainan, China	Firstly sequenced and assembled in this study
	NG96-14	Papua New Guinea	Firstly sequenced and assembled in this study
	Christalin	Unknown	Firstly sequenced and assembled in this study
S. hybird	GT42	Guangxi, China	Firstly sequenced and assembled in this study
	POJ2878	Java, Indonesia	Firstly sequenced and assembled in this study
	ZhongzheNo1	Guangxi, China	Firstly sequenced and assembled in this study
	ROC25	Taiwan, China	Firstly sequenced and assembled in this study
N. porphyrocoma	HebawangNo1	Guangxi, China	Firstly sequenced and assembled in this study
T. arundinaceum	BM87-36	Guangxi, China	Firstly sequenced and assembled in this study

Table S2 The List of genes in the chloroplast genomes of Saccharinae species were assembled in this study.

Category of genes	Group of genes	Name of genes
Photosynthesis genes	Subunits of photosystem I	*psaA, psaB, psaC, psaI, psaJ*
	Subunits of photosystem II	*psbA, psbB, psbC, psbD, psbE, psbF, psbI, psbJ, psbK, psbL, psbM, psbN, psbT, psbZ, ycf3*
	Subunits of Cytochrome b6/f/complex	*petA, petB, petD, petG, petL, petN*
	Subunits of ATP synthase	*atpA, atpB, atpE, atpF, atpH, atpI*
	Subunits of Rubisco	*rbcL*
	Subunits of NADH-dehydrogenase	*ndhA, ndhB*$^{\times2}$*, ndhC, ndhD, ndhE, ndhF, ndhG, ndhH, ndhI, ndhJ, ndhK*
Self-replicating genes	Large subunit ribosomal proteins	*rpl14, rpl16, rpl2*$^{\times2}$*, rpl20, rpl22, rpl23, rpl32, rpl33, rpl36*
	Small subunit ribosomal proteins	*rps11, rps12*$^{\times2}$*, rps14, rps15*$^{\times2}$*, rps16, rps18, rps19*$^{\times2}$*, rps2, rps3, rps4, rps7*$^{\times2}$*, rps8*
	DNA dependent RNA polymerase	*rpoA, rpoB, rpoC1, rpoC2*
	Ribosomal RNA genes	*rrn16S*$^{\times2}$*, rrn23S*$^{\times2}$*, rrn4.5S*$^{\times2}$*, rrn5S*$^{\times2}$
	Transfer RNA genes	*trnA-UGC*$^{\times2}$*, trnC-GCA, trnD-GUC, trnE-UUC, trnF-GAA, trnG-GCC, trnG-UCC, trnH-GUG*$^{\times2}$*, trnI-CAU*$^{\times2}$*, trnI-GAU*$^{\times2}$*, trnK-UUU, trnL-CAA*$^{\times2}$*, trnL-UAA, trnL-UAG, trnM-CAU*$^{\times2}$*, trnN-GUU*$^{\times2}$*, trnP-UGG, trnQ-UUG, trnR-ACG*$^{\times2}$*, trnR-UCU, trnS-GCU, trnS-GGA, trnS-UGA, trnT-GGU, trnT-UGU, trnV-GAC*$^{\times2}$*, trnV-UAC, trnW-CCA, trnY-GUA*
Other genes	Envelop membrane protein	*cemA*
	Translational initiation factor	*infA*
	c-type cytochrome synthesis gene	*ccsA*
	ATP-dependent/Protease	*clpP*
	Maturase	*matK*
Unknown function protein-coding gene		*ycf4*
